# Orchestration of an Uncommon Maturation Cascade of the House Dust Mite Protease Allergen Quartet

**DOI:** 10.3389/fimmu.2014.00138

**Published:** 2014-03-31

**Authors:** Marie-Eve Dumez, Julie Herman, Vincenzo Campizi, Moreno Galleni, Alain Jacquet, Andy Chevigné

**Affiliations:** ^1^Laboratory of Retrovirology, Department of Infection and Immunity, Centre de Recherche Public Santé, Luxembourg, Luxembourg; ^2^Macromolécules Biologiques, Department of Life Sciences, Centre for Protein Engineering, University of Liège, Liège, Belgium; ^3^Faculty of Medicine, Department of Medicine, Division of Allergy and Clinical Immunology, Chulalongkorn University, Bangkok, Thailand

**Keywords:** mite, proteases, Der p 1, allergen, activation cascade, localization, interaction

## Abstract

In more than 20% of the world population, sensitization to house dust mite allergens triggers typical allergic diseases such as allergic rhinitis and asthma. Amongst the 23 mite allergen groups hitherto identified, group 1 is cysteine proteases belonging to the papain-like family whereas groups 3, 6, and 9 are serine proteases displaying trypsin, chymotrypsin, and collagenolytic activities, respectively. While these proteases are more likely to be involved in the mite digestive system, they also play critical roles in the initiation and in the chronicity of the allergic response notably through the activation of innate immune pathways. All these allergenic proteases are expressed in mite as inactive precursor form. Until recently, the exact mechanisms of their maturation into active proteases remained to be fully elucidated. Recent breakthroughs in the understanding of the activation mechanisms of mite allergenic protease precursors have highlighted an uncommon and unique maturation pathway orchestrated by group 1 proteases that tightly regulates the proteolytic activities of groups 1, 3, 6, and 9 through complex intra- or inter-molecular mechanisms. This review presents and discusses the currently available knowledge of the activation mechanisms of group 1, 3, 6, and 9 allergens of *Dermatophagoides pteronyssinus* laying special emphasis on their localization, regulation, and interconnection.

## Introduction

House dust mites (HDMs; *Dermatophagoides* spp.) are common reservoirs of potent airborne allergens, which induce Th2-biased inflammatory diseases such as allergic asthma, perennial rhinitis as well as atopic dermatitis in sensitized patients (1). To date, over 23 different HDM allergen groups inducing the production of allergen-specific IgE in humans have been referenced (2).

A growing amount of literature suggests that HDM allergens can stimulate numerous innate immune activation pathways to initiate the Th2 allergic response (3). Although HDM allergens can induce lung inflammation by protease-independent mechanisms, the proteolytic activities of HDM allergens trigger key innate signaling to initiate the allergic response through, among others, the disruption of the airway/skin epithelial barrier, the protease-activated receptor-2 (PAR-2) activation, and other cell-surface receptor cleavages (3–5). These proteolytic attacks facilitate the uptake of the allergens by dendritic cells (DCs) in subepithelial tissues and lead to the release of numerous proinflammatory (IL-6, IL-8, and IL-1β) as well as innate Th2 (IL-25, IL-33, and TSLP) cytokines from the target cells. While the crystal structure of Der p 1 demonstrated that this allergen is a papain-like cysteine protease, sequence homologies, and protease inhibition assays proved that Der p 3, Der p 6, and Der p 9 belong to the trypsin-like, chymotrypsin-like, and collagenolytic-like serine protease families, respectively (6). Although the biological roles of these proteases in mites have not hitherto been completely unraveled, these allergens could more probably play a digestive function for the mite as they were detected in the gut as well as in mite feces. The four HDM allergen proteases are all synthesized as pre-zymogens formed by a signal peptide essential for the secretion, an N-terminal propeptide followed by the mature protease domain. Each corresponding prosequence inhibits the respective protease to prevent cellular toxicity during their expression. Considering the critical role of proteolytically active HDM allergens in the initiation of the allergic response, the elucidation of the pathways for the maturation of these allergens offers opportunities to deeply characterize their proteolytic specificities allowing the identification of their corresponding protein substrates on the target innate and adaptive immune cells.

The present minireview will update the information about the inter- and intra-molecular maturation mechanisms of the protease allergens from *Dermatophagoides pteronyssinus* with special emphasis on particular features of propeptides and protease interactions. We will highlight that the HDM protease quartet processing follows an uncommon and interconnected maturation pathway, which is uniquely orchestrated by Der p 1.

## The Maturation of Allergen Proteases from *Dermatophagoides pteronyssinus*

### proDer p 1

Mite cysteine protease Der p 1 (group 1) belongs to the papain-like protease family (CA1) and is considered one of the most potent HDM allergens on the basis of the high frequency (70–100%) of specific IgE in HDM allergic patients (7, 8) as well as of its capacity to proteotically trigger innate immune activation (3). Through the removal of its signal peptide (18 residues), Der p 1 is secreted as an inactive zymogen, proDer p 1, composed of a catalytic domain of 222 residues and an N-terminal propeptide of 80 residues, which acts as an internal chaperone during protease folding and then locks the protease active site (9, 10). The crystallographic structure of proDer p 1 revealed that the propeptide of Der p 1 adopts a unique fold within the CA1 protease family (10). The propeptide of Der p 1 notably displays an intermediate size (80 residues) and is devoid of the canonical ERFNIN motif in its N-terminal globular domain (Table 1). The propeptide of Der p 1 is also characterized by the presence of an additional fourth α-helix replacing the unstructured C-terminal tail normally found in the other propeptide subfamilies (60 or 100 residues).

**Table 1:** **Propeptides of *Dermatophagoides pteronyssinus* proteases**.

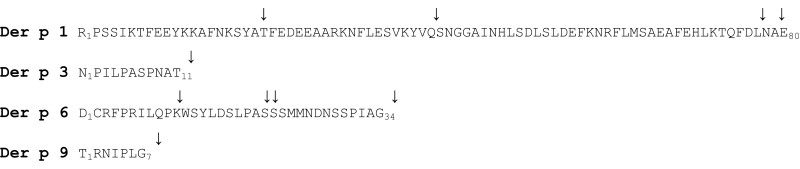

*The arrows indicate the identified Der p 1 cleavage sites*.

*In vitro* activation of proDer p 1 produced by the yeast *Pichia pastoris* or by S2 insect cells was shown to be induced under acidic conditions (i.e., pH 4) (11–14). Biophysical studies demonstrated that under acidic conditions, the propeptide of Der p 1 partly unfolds, leading to a considerable increase in the solvent accessibility and flexibility of the residues located in the N-terminal globular domain. Under these conditions, the propeptide loses its inhibitory ability and becomes a substrate for Der p 1 and most probably for other mite allergen proteases (11, 15–17). *In vitro*, the auto-activation of proDer p 1 at pH 4 leads to the formation of intermediates, which correspond to the successive loss of the first and second N-terminal α-helices following cleavages at the -NKS*Y_19_-A_20_*TFE- and -KYV*Q_40_-S_41_*NGG- sites, respectively, considering the first residue of the zymogen as residue 1 (11, 13, 18) (Table 1). Generation of fully active Der p 1 with or without two additional residues (AE_80_) is then achieved through a final cleave at overlapping cleavage sites (-FDL*N_78_-A_79_*ETN- or -LNA*E_80_-T_81_*NAC-) located at the propeptide C-terminus (11, 13, 18–20). It is noteworthy that these cleavages take place in propeptide regions that correspond to solvent exposed coil connecting the different α-helices and at sequences corresponding to Der p 1 proteolytic specificity (21). Activation of proDer p 1 was also shown to occur through inter-molecular cleavages of the precursor by active Der p 1 protease (14, 15). The proDer p 1 sequence contains two N-glycosylation sites, one within the propeptide (-*N_16_*KS-) and one within the catalytic domain (-*N_132_*QS-) the latter being glycosylated in the recombinant and natural forms of Der p 1 (11, 13, 14, 22, 23). Surprisingly, while pH is the major factor triggering proDerp 1 maturation, the glycosylation of the Der p 1 propeptide by the yeast *P. pastoris* at Asn16, which is N-terminally located to the -*N_16_*KS*Y_19_-A_20_*TFE- cleavage site, was shown to decelerate the activation rate of the zymogen (11, 24). Although the glycosylation pattern of the Der p 1 precursor in mites is most probably different, one cannot rule out that such interference might also be observed in mites and could consequently constitute a regulation system for allergen maturation.

### proDer p 3

Based on the high percentage of its sequence identity with trypsin-like enzymes but also on its proteolytic specificity (i.e., preference for an Arg or a Lys residue in P1 position), Der p 3 (group 3) was classified into the S1A serine protease family (25–27). To date, the binding of IgE from sera of allergic patients to Der p 3 appears controversial and varies between 10 and 100% (7, 25, 27–30). Although the protein substrates targeted by Der p 3 still need to be fully elucidated, the proteolytic activation of PAR-2 by Der p 3 was clearly demonstrated (31, 32). Moreover, the enzymatic activity of a recombinant form of Der p 3 toward the QAR-AMC fluorescent peptide substrate was demonstrated to be 50 times higher than that of Der p 1, thereby indicating that although present in low quantity in the HDM extracts, Der p 3 greatly contributes to the total proteolytic activity of the HDM extracts (33).

Der p 3 is synthesized in mites as a pre-zymogen constituted of a signal peptide (18 residues), a propeptide of 11 residues, and a serine protease domain of 232 residues (Table 1) (25). In contrast to the Der p 1 propeptide, the Der p 3 prosequence was shown as not involved in the correct folding of the zymogen (34). Although it shows poor inhibitory capacity toward the mature protease, the Der p 3 propeptide is essential to block the Ile_12_ residue of Der p 3 and to maintain the allergen in less active conformation as previously observed for trypsinogen, the precursor of trypsin (33–35). Trypsinogen is commonly activated through inter-molecular cleavages by the membrane serine protease enterokinase, following the recognition of a conserved poly-aspartyl lysine motif [(D)DDDK] located at the end of the propeptide (36, 37). Alternatively, trypsinogen can be activated through autocatalytic cleavage occurring after neutralization of the negative charges of the poly-aspartyl lysine motif by calcium ions (36, 38, 39). Compared with other trypsin proteases, the propeptide of proDer p 3 (N*P*IL*P*AS*P*NA*T_11-_*) shows some distinct features such as the presence of a Thr instead of an Arg or a Lys residue in P1 position. Consequently and in contrast to trypsinogen, no auto-activation of recombinant proDer p 3 was observed (33). The activation mechanism of proDer p 3 produced in *P. pastoris* was shown to be inter-molecular and led by cysteine protease Der p 1 (33) (Table 1). It is noteworthy that the N-glycosylation at -*N_9_*AT_11_- site within the propeptide decreased the maturation rate as observed for proDer p 1 (33). The maturation of proDer p 3 was also shown to depend on the interactions between the polyproline motif (*P_2_*IL*P_5_*AS*P_8_*) of the propeptide and Der p 1, since mutation or deletion in this motif, especially of Pro_5_ and Pro_8_, drastically reduced the activation rate of the zymogen (33, 34). This uncommon polyproline motif within the protease propeptide was also demonstrated to protect proDer p 3 against undesired hydrolysis (33, 34). Indeed, as observed for trypsin, mature Der p 3 undergoes rapid autolysis through cleavages at the -GGE*K_17_*-*A_18_*LAG- and -KNA*K_115_*-*A_116_*VGL- sites, explaining more probably the low amount of Der p 3 detected in HDM extracts (25, 27, 33, 34).

### proDer p 6

Der p 6 (group 6) is a chymotrypsin-like serine protease (S1 family) that preferentially cleaves peptide bonds preceded by an aromatic residue (i.e., Phe, Tyr, and Trp) (30). The precursor of Der p 6 is composed of a signal peptide of 16 residues, a propeptide of 34 amino acids, and a catalytic domain of 231 amino acids (Table 1) (40). The propeptide of Der p 6 has recently been shown to act as an inhibitor of the cognate catalytic domain (16). This suggested that as for Der p 1 and Der p 3, the propeptide of Der p 6 regulates the spatio-temporal activation of the protease zymogen in mites (16). Similarly to Der p 3, the propeptide of Der p 6 was shown as not required for the correct folding of recombinant Der p 6 expressed in *P. pastoris* (16).

Surprisingly, while chymotrypsinogen displays an Arg at the C-terminus of its propeptide for the recognition and cleavage by trypsin (41, 42), the C-terminal extremity of the Der p 6 propeptide (-*P_31_*I*A_33_*G-) is highly similar to that of the Der p 3 propeptide (-*P_8_*N*A_10_*T-). In line with this observation, we recently showed that as for proDer p 3, proDer p 6 can be activated by Der p 1 providing a fully active Der p 6 protease presenting the expected mature N-terminal extremity (*V_35_*IGG-) (Table 1) (16).

### proDer p 9

Although very poorly characterized, Der p 9 (group 9) is classified as a collagenolytic-like serine protease on the basis of its ability to hydrolyze collagen (43). Interestingly, its high percentage of identity with Der p 3 (76%) together with the conservation of the residues corresponding to the catalytic triad (His_48_–Asp_88_–Ser_200_) as well as those related to the specificity pocket all suggest that Der p 9 could be a trypsin-like protease. Moreover, like Der p 3 and trypsin, Der p 9 was shown to activate PAR-2 through a proteolytic cleavage occurring at the -SKG*R_36_-S_37_*LIG- site of the receptor (32).

The Der p 9 pre-zymogen is composed of a signal peptide of 17 residues, a propeptide of 7 amino acids (TRNIPL*G_7_*_−_) preceding a 220-residue catalytic domain (Table 1) (43) (Uniprot: Q8MWR5). The role of the propeptide and the activation mechanism leading to fully active protease Der p 9 remain to be fully elucidated. By using fluorescence resonance energy transfer (FRET) substrates, we have recently demonstrated that recombinant and natural active Der p 1 cleave the peptide mimicking the junction between the propeptide and the mature form of Der p 9 (Dnp-IPL*G_7_*-*V_8_*IGG-AMC), which suggests that Der p 1 could also be critical for the maturation of proDer p 9 (Table 1) (16). Nevertheless, the isolation of another cDNA coding for a Der p 9 related serine protease with an alternative putative extended propeptide sequence (Uniprot: Q7Z163, Q8MWR4) would require additional experiments.

## Uncommon and Unique Activation Pathway

**Figure 1:**
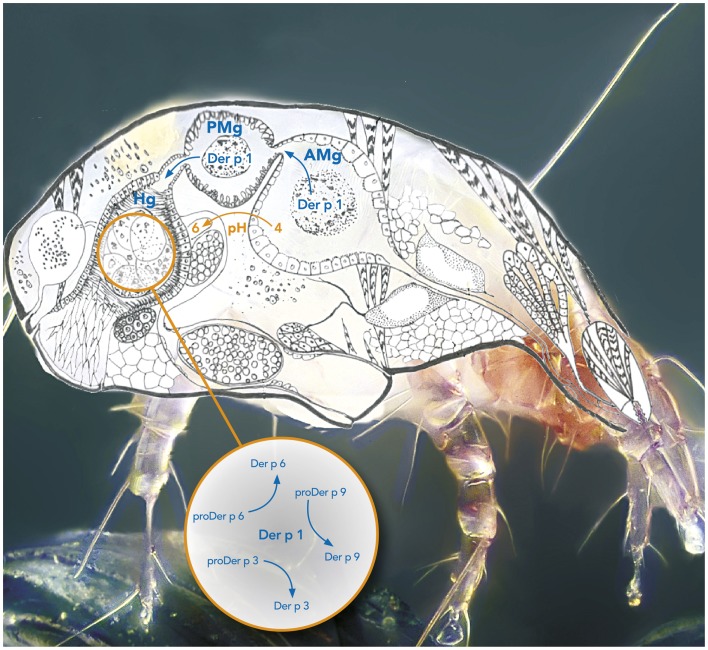
**Protease activation cascade in the digestive tract of the mite Dermatophagoides pteronyssinus**. The box represents the activation cascade in the hindgut. AMg, Anterior midgut; PMg, Posterior midgut; Hg, Hindgut.

Taken together, the *in vitro* results generated using recombinant forms of the different zymogens and FRET substrates clearly demonstrate the major role of Der p 1 in the activation process of the *D. pteronyssinus* mite allergen proteases (11, 13, 14, 16, 18, 33). Following its auto-activation under acidic conditions, Der p 1 remarkably orchestrates the inter-molecular maturation of its own precursor (proDer p 1) but also of serine protease precursors proDer p 3, proDer p 6, and most probably proDer p 9 (Figure 1) (11, 13, 14, 16, 18, 33).

Although the exact location where the maturation of the allergen proteases takes place in the mite remains unknown, different hypotheses can be considered. Mite proteases Der f 1, Der p 1, Der f 3, and Der p 6 were all immuno-localized in the digestive tract of the *Dermatophagoides farinae* and *D. pteronyssinus* species (16, 44–46). In particular, Der p 1 was localized in the cells lining the anterior midgut (AMg) corresponding to an acidic environment (pH 4), in the posterior midgut (PMg) (pH 5) as well as in the hindgut (Hg) where the pH was shown to reach a value of 6 (16, 44, 45, 47, 48). It is therefore plausible that proDer p 1 is secreted in the anterior gut and activated in the acidic lumen. Alternatively, it is worth noticing that the Der p 1 propeptide contains a highly conserved two-lysine motif (Lys_37_ and Lys_72_) that might be involved in the targeting of the zymogen to the acidic vesicles of the anterior gut cells to initiate its intracellular maturation before its release in the lumen (49, 50). Serine proteases Der f 3 and Der p 6 were observed in the Hg (16, 46) and Der p 1 was co-localized with Der p 6 in the Hg of *D. pteronyssinus* sections indicating that mature protease Der p 1 could activate the secreted serine protease zymogens in the Hg where pH corresponds to its maximum activity (i.e., pH 6.5) (16, 48).

The activation mechanisms of the serine protease zymogens of the trypsin-like family (proDer p 3 and proDer p 6) by a cysteine protease (Der p 1) appear to be very uncommon for such protease families and are most probably related to the presence of specific residues at the C-termini of the propeptides. Noticeably, the P4–P3–P2–P1 residues (Schechter and Berger nomenclature) N-terminally located to the cleavage sites of the Der p 1 (*L*N*A*E_80_-), Der p 3 (*P*N*A*T_11_-), Der p 6 (*P*I*A*G_34_-), and Der p 9 (*I*PLG_7_-) proteases are all similar and perfectly match Der p 1 specificity (21). It is noteworthy that the propeptides of homologous zymogens from other dust mites such as the *D. farinae* and *Euroglyphus maynei* species exhibit a high degree of similarity to those from *D. pteronyssinus* suggesting that a similar proteolytic pathway might also occur in these organisms (Table 2) (16).

**Table 2:** **Activation sites of zymogens from *Dermatophagoides pteronyssinus, Dermatophagoides farinae*, and *Euroglyphus maynei* species**.

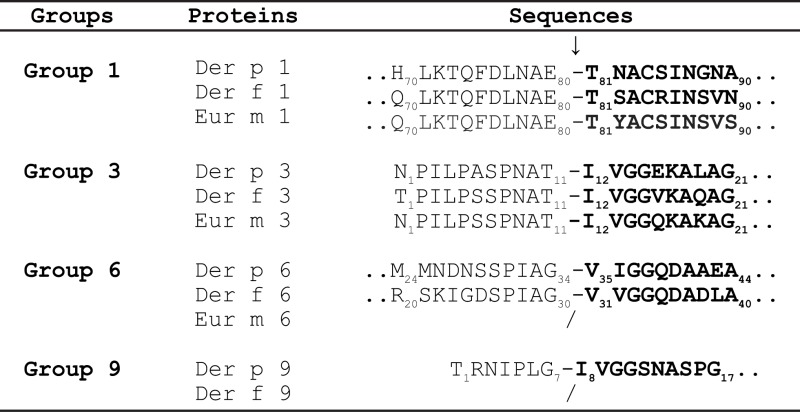

*The arrow indicates the putative cleavage sites between the propeptide and the mature protease sequences which are in bold. (/) Unknown sequences*.

## Conclusion

During the last decade, we and others have unraveled the *in vitro* activation mechanisms of the mite cysteine (Der p 1) and serine (Der p 3, Der p 6, and Der p 9) protease precursors. All the generated data highlighted the role of Der p 1 as the “maestro” in the maturation processes of the different HDM protease allergens. This orchestration which appears rather uncommon among the protease world depends on specific sequences present at the C-terminus of the different propeptides.

Although it remains to be demonstrated that *in vivo* HDM protease allergen maturation is similar to the *in vitro* observations, the elucidation of the present activation cascade firstly provides key information for the design of new potent specific inhibitors to these clinically relevant allergens. Such molecules represent potential novel acaricidal compounds to control the HDM population by impairing their digestive function. The critical role of PAR-2 activation in HDM allergy and the effective PAR-2 cleavage by at least Der p 3 and Der p 9 demonstrates the interest in the blockage of the proteolytic activity to modulate the HDM allergic response (31, 32, 51, 52). It must be pointed out that the first preclinical results generated with inhaled Der p 1-specific allergen delivery inhibitors also provide clear evidence for the interest of such therapeutics in the treatment of HDM allergy (53).

Secondly, consistent productions of highly pure and fully active recombinant mature HDM protease allergens could open the way for further characterization of their proteolytic specificities, for better definition of their respective interplay with the innate and adaptive immune system and for analysis of their IgE reactivity. Finally, the mapping of their corresponding IgE-binding epitopes, in the absence of any propeptide interference (epitope masking), could initiate the development of hypoallergenic variants for novel immunotherapeutic treatments.

## Author Contributions

Marie-Eve Dumez and Andy Chevigné designed the content and supervised the writing of the publication. Marie-Eve Dumez, Julie Herman, Vincenzo Campisi, Alain Jacquet, and Andy Chevigné wrote the minireview. Moreno Galleni critically revised the intellectual content of the publication. All authors gave their final approval to the version to be published.

## Conflict of Interest Statement

The authors declare that the research was conducted in the absence of any commercial or financial relationships that could be construed as a potential conflict of interest.
